# Artificial Intelligence-Guided Inverse Design of Deployable Thermo-Metamaterial Implants

**DOI:** 10.1021/acsami.4c17625

**Published:** 2025-01-02

**Authors:** Pengcheng Jiao, Chenjie Zhang, Wenxuan Meng, Jiajun Wang, Daeik Jang, Zhangming Wu, Nitin Agarwal, Amir H. Alavi

**Affiliations:** † Ocean College, Zhejiang University, Zhoushan 316021, China; ‡ Department of Bioengineering, University of Pittsburgh, Pittsburgh, Pennsylvania 15261, United States; § Department of Mechanical Engineering and Materials Science, University of Pittsburgh, Pittsburgh, Pennsylvania 15261, United States; ∥ Department of Civil and Environmental Engineering, University of Pittsburgh, Pittsburgh, Pennsylvania 15261, United States; ⊥ College of Engineering, Cardiff University, Cardiff CF10 3AT, U.K.; # Department of Neurological Surgery, University of Pittsburgh School of Medicine, Pittsburgh, Pennsylvania 15213, United States; ¶ Department of Neurological Surgery, University of Pittsburgh Medical Center, Pittsburgh, Pennsylvania 15213, United States; ∇ Neurological Surgery, Veterans Affairs Pittsburgh Healthcare System, Pittsburgh, Pennsylvania 15240, United States

**Keywords:** thermal mechanical metamaterials, medical implants, inverse design, artificial intelligence

## Abstract

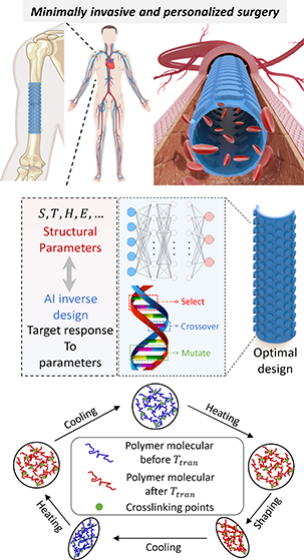

Current limitations in implant design often lead to trade-offs between minimally invasive surgery and achieving the desired post-implantation functionality. Here, we present an artificial intelligence inverse design paradigm for creating deployable implants as planar and tubular thermal mechanical metamaterials (thermo-metamaterials). These thermo-metamaterial implants exhibit tunable mechanical properties and volume change in response to temperature changes, enabling minimally invasive and personalized surgery. We begin by generating a large database of corrugated thermo-metamaterials with various cell structures and bending stiffnesses. An artificial intelligence inverse design model is subsequently developed by integrating an evolutionary algorithm with a neural network. This model allows for the automatic determination of the optimal microstructure for thermo-metamaterials with desired performance,i.e., target bending stiffness. We validate this approach by designing patient-specific spinal fusion implants and tracheal stents. The results demonstrate that the deployable thermo-metamaterial implants can achieve over a 200% increase in volume or cross-sectional area in their fully deployed states. Finally, we propose a broader vision for a clinically informed artificial intelligence design process that prioritizes biocompatibility, feasibility, and precision simultaneously for the development of high-performing and clinically viable implants. The feasibility of this proposed vision is demonstrated using a fuzzy analytic hierarchy process to customize thermo-metamaterial implants based on clinically relevant factors.

## Introduction

The field of biomedical engineering is undergoing a transformation with the emergence of mechanical metamaterials. Engineered with unique properties beyond those found in nature, these materials open exciting possibilities for patient-specific implants.^1^ Mechanical metamaterials exhibit characteristics such as a negative Poisson’s ratio,^2^ negative compression,^3^ and negative thermal expansion.^4^ Additionally, their microstructures can be rationally designed with responsive materials and as composite systems, enabling advanced functionalities beyond the mechanical domain, including sensing,^5^ programmability,^6^ magnetic actuation,^7^ and shape memory effect.^8^ Traditional materials used for clinical implants, such as metals and ceramics, are inherently stiff and lack the intricate, adaptive structures found in natural tissues. This rigidity can cause stress shielding in specific applications (e.g., bone healing), where the implant absorbs most of the load.^9,10^ Unlike traditional implants with fixed properties, mechanical metamaterials can be tailored to an individual’s specific needs due to their tunable performance. This customization optimizes factors like stiffness and porosity to match the surrounding tissue.^11^ Furthermore, advancements in additive manufacturing technology allow for the creation of these complex structures directly during surgery. This capability can potentially eliminate the need for premanufactured implants and offer customization and convenience for patients and surgeons.^1^ A notable innovation within this field is the creation of deployable mechanical metamaterial implants.^12,13^ In their compact form, these implants can be delivered to the surgical site through minimally invasive procedures.^13^ Such implants require smaller incisions and minimize patient discomfort and recovery time. Once in place, they can expand to their functional size. Deployable metamaterial implants can be made from different shape memory polymers such as polylactic acid (PLA), poly(glycerol sebacate) (PGS), poly(ε-caprolactone) (PCL), poly(ethylene glycol) (PEG)-based hydrogels, etc. The unique properties of these polymers within deployable metamaterial implant frame could provide a significant advantage in surgical applications.^13^

However, a major challenge lies in designing these intricate implantable structures. Unit cells, the building blocks of mechanical metamaterials, can be configured with diverse sizes, dimensions, and shapes. This results in an expansive design space that is impractical to navigate manually or explore through experimental testing. Arguably, optimizing the structure and performance of these implants for specific applications requires a powerful approach. Artificial intelligence (AI) emerges as a viable solution for this complex problem.^14−18^ Conventional AI methods, such as neural networks, have recently been used to predict the performance of mechanical metamaterials like octet truss metamaterials,^19^ curved beams,^20^ and lattice structures.^21^ More recently, AI inverse design has been applied to create specific mechanical metamaterials with predefined performance characteristics for applications such as energy absorption,^22,23^ smart soles,^24^ and soft robots.^25^ While AI methods offer remarkable potential for exploring the design space of mechanical metamaterials, their application in designing implantable systems, especially deployable implants, remains largely unexplored. Deployable implants are particularly important for this purpose because traditional implants may not possess the necessary mechanical properties once deployed in the body. Conversely, sturdier implants that offer the desired properties often require larger incisions, leading to increased patient recovery time and discomfort.

Here, we introduce an AI-guided inverse design approach to develop deployable implants in the form of planar and tubular thermal mechanical metamaterials (thermo-metamaterials) for minimally invasive and personalized surgery. Initially, corrugated thermo-metamaterials featuring square and hexagonal cells with different structural parameters and bending stiffnesses are designed. The created database of corrugated thermo-metamaterials is used to construct a neural network that can accurately predict their bending stiffnesses. Then, an AI inverse design model is proposed by integrating an evolutionary process to the network to derive microstructural parameter sets for thermo-metamaterials with desired bending stiffnesses. We validate the effectiveness of the proposed approach by designing deployable spinal fusion implants and lung stents. Finally, we present a novel vision for a clinically informed AI inverse design approach that prioritizes biocompatibility, feasibility, and precision for successful clinical translation.

## Results and Discussion

**Figure 1:**
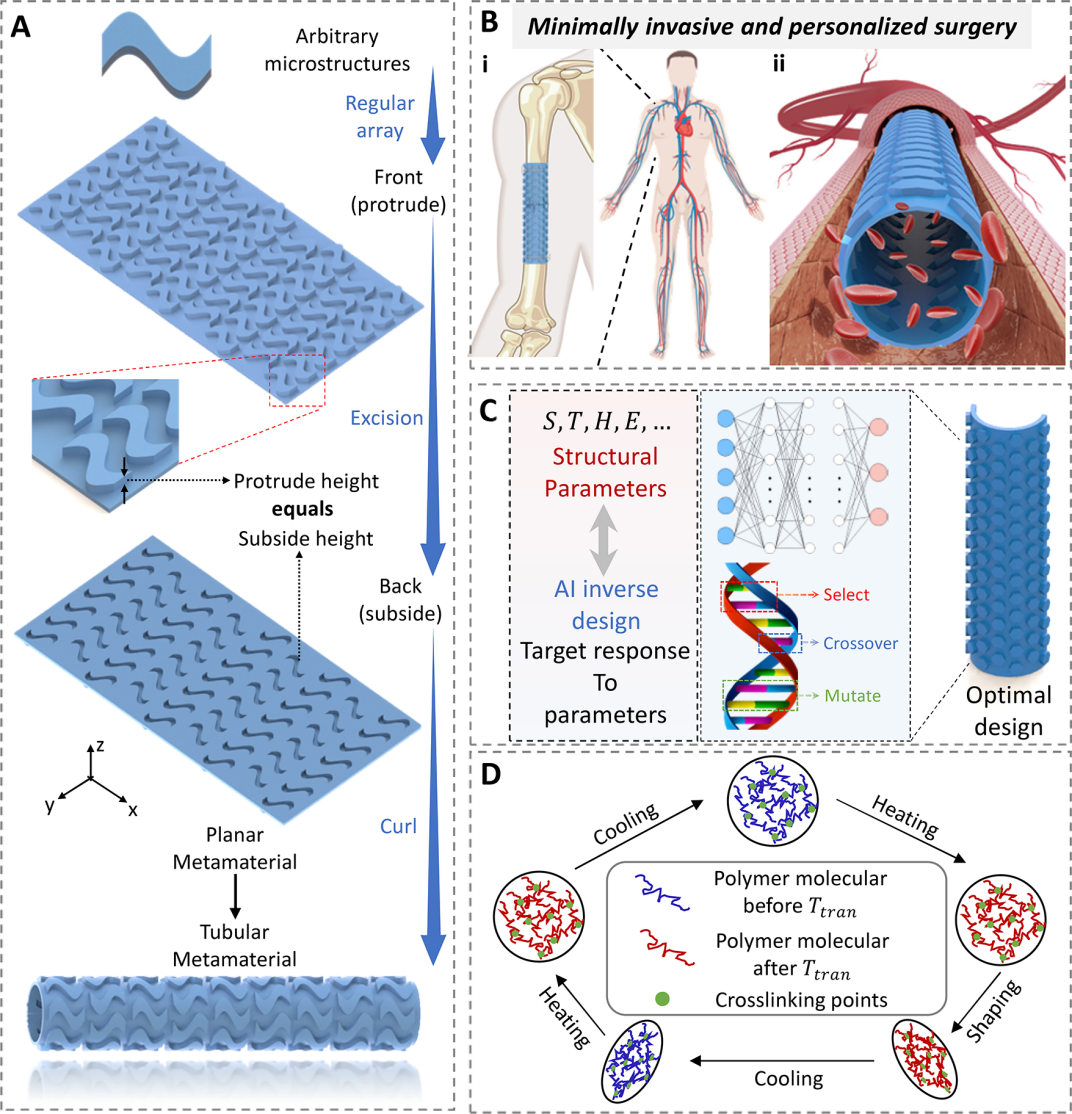
Vision for designing deployable implants using an AI inverse design approach. (A) Corrugated metasurfaces are 2D counterparts of corrugated mechanical metamaterials. Engineered with customized microstructures and patterned with periodic surface corrugations, they achieve specific mechanical characteristics, in particular tunable BS. Biocompatible shape memory polymers can be used to create these metasurfaces, which can then be rolled into tubular configurations to form tubular thermo-metamaterials. (B) Potential applications of corrugated metasurfaces with tunable *BS* include their use in minimally invasive and personalized surgery. These metasurfaces can fashioned in either planar or tubular configurations to fabricate thermo-metamaterial humeral fracture implants or vascular scaffolds. These implants can be designed to respond to changes in body temperature or externally induced temperature variations, expanding or contracting as needed, thus facilitating their insertion through small incisions with minimal invasiveness. Once deployed, they assume their predetermined functional shape within the body. (C) Accelerating the design and discovery of thermo-metamaterials via an AI inverse design approach. Integrating clinically informed criteria into the AI inverse design process ensures the feasibility, precision and biocompatibility of the explored designs. (D) The deployment process of the thermo-metamaterial implants 4D printed using shape memory polymers. This process consists of heating the thermo-metamaterial structure to its pliable state, deforming it for insertion, cooling it and finally reheating it to regain its original shape.

Here, we define thermo-metamaterials as mechanical metamaterials tailored to exhibit unique mechanical responses to thermal stimuli. This unique characteristic allows them to be used as deployable implants. Thermo-metamaterial implants can be designed to expand or contract in response to body temperature or externally induced temperature variations, allowing for minimally invasive insertion through a small incision and subsequent deployment to their functional shape within the body. This adaptability can potentially simplify surgical procedures and enhance the performance and integration of the implant within biological tissues. Figure 1 illustrates the overarching vision of this research, wherein we employ an AI approach for the inverse design of thermo-metamaterial implants. We consider corrugated metasurfaces to design thermo-metamaterial implants with tunable bending stiffness (BS) (Figure 1A). Corrugated metasurfaces are essentially 2D counterparts of corrugated mechanical metamaterials with periodic surface corrugations designed to obtain desired properties.^26−29^ These metasurfaces can be utilized in planar form to design plate-like implants such as humeral fracture implants to stabilize and support the healing of bone fractures. Alternatively, they can be rolled to create tubular metamaterials, serving as stents for hollow organs such as the lungs, esophagus, or blood vessels (Figure 1B). From a structural standpoint, corrugated metasurfaces can be rationally designed to provide substantially larger BS compared to conventional plates of the same thickness, while also offering opportunities for more versatile structural configurations. For instance, preliminary tests conducted in this study demonstrate that a proof-of-concept corrugated metasurface exhibits approximately 55.7% higher BS than a plain plate (see Figure S1 in the Supporting Information).

However, a major challenge in designing such metasurfaces lies in the intricate process of precisely tailoring the shape, size, and periodicity of the corrugations. This process involves navigating a vast array of potential configurations. The design and discovery of these configurations can be facilitated by an AI-powered inverse design approach that integrates a standard neural network with an evolutionary computational process (Figure 1C). The inverse design algorithm can generate designs for implantable devices with a targeted mechanical response informed by physicians’ recommendations. These designs are then modeled and fabricated, facilitating personalized surgery tailored to individual patient needs. For a material perspective, thermo-metamaterial implants can be made from biocompatible shape memory polymers and 4D printed using additive manufacturing techniques, such as stereolithography, fused deposition modeling, or selective laser sintering, to create deployable implants. 4D printing involves materials that can self-transform or self-assemble over time when subjected to external stimuli like heat, moisture, or light, adding a temporal dimension to the 3D printing process. The deployment process involves four steps (Figure 1D): first, the thermal metamaterial is heated above its transition temperature to become pliable (initial configuration); second, it is deformed into a compact configuration for easy insertion (deforming configuration); third, it is cooled below this temperature while maintaining its shape to lock in the temporary form (programming process); finally, once in place, the material is reheated above the transition temperature, allowing it to return to its original, functional shape (recovery process). This entire shape transformation process can be theoretically formulated for a precise and efficient deployment of the implant within the body.

### Thermo-Metamaterials with Various Corrugations

**Figure 2:**
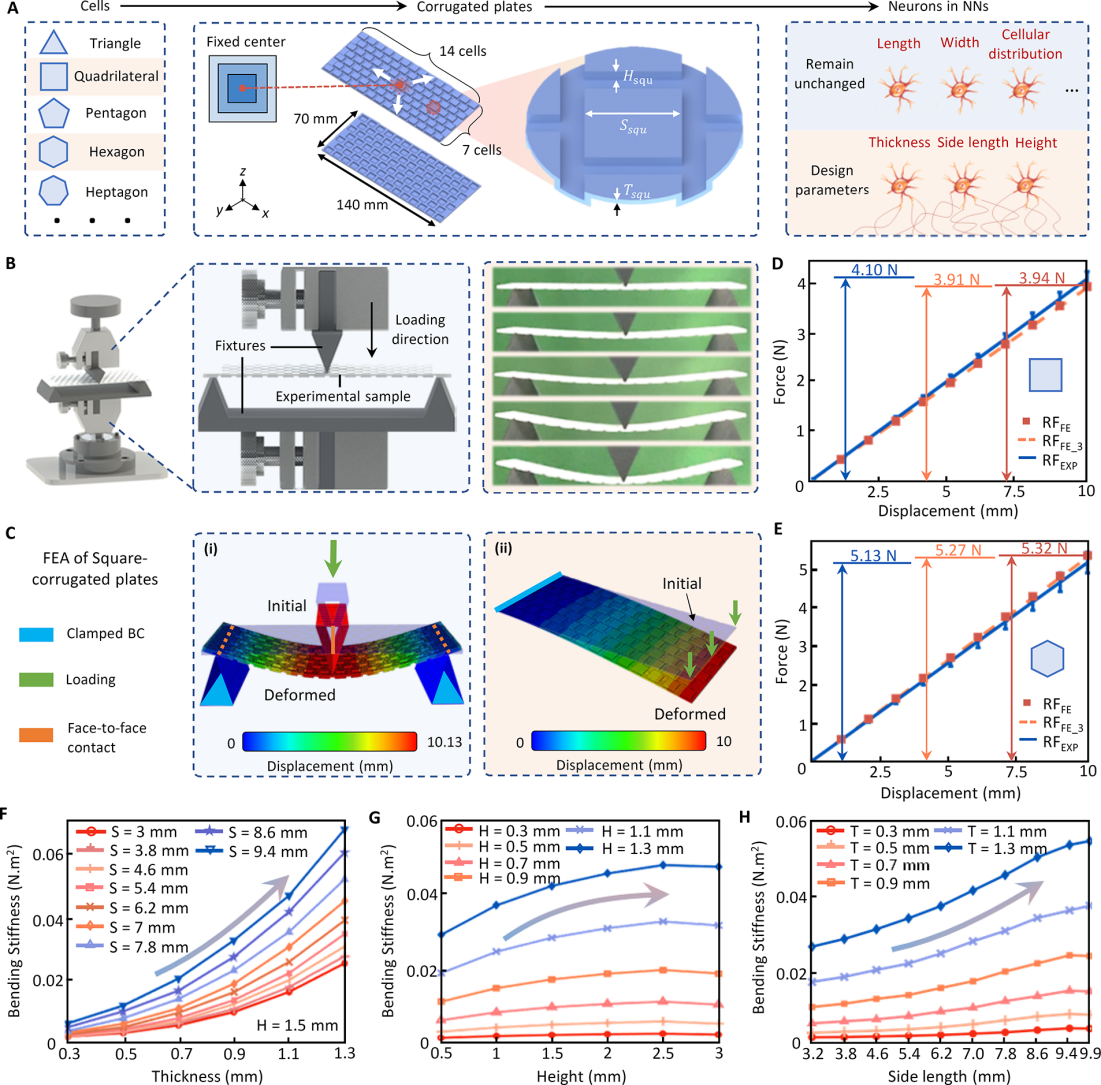
Design of microstructures and generation of data set for planar thermo-metamaterials. (A) Planar thermo-metamaterials with various corrugation patterns. (B) Experimental testing for the BS and out-of-plane deformation procedures. (C) Numerical simulations of planar thermo-metamaterials with square corrugations. Comparisons of the experimental and numerical results on the planar thermo-metamaterials with the (D) square and (E) hexagonal corrugations. Bending stiffness variations of the planar thermo-metamaterials with respect to the (F) thickness *T*, (G) height *H* and (H) side length *S*.

To investigate the design possibilities for thermo-metamaterials, we first examine planar structures in the form of corrugated metasurfaces. These metasurfaces feature various corrugation patterns, including triangular, square, rectangular, pentagonal, hexagonal, and heptagonal configurations, as depicted in Figure 2A. We maintained constant length, width, cellular distribution, and other parameters of the plate. Additionally, PLA with an elastic modulus (*E*) of 2400 MPa was used to fabricate the specimens. This resulted in three tunable parameters for the planar thermo-metamaterials: the thickness (*T*) of the plate, the height (*H*) of the cell, and the side length (*S*) of the cells. Three-point bending experiments were conducted to obtain the BS of the planar thermo-metamaterials (Figure 2B). The upper and lower fixations were clamped on the fatigue test machine, the sample was placed flat on the lower fixation, and the upper fixation was loaded in the middle position of the corrugated plate. Details of the 4D printing fabrication and testing are presented in Materials and Methods. Next, we develop the numerical models to investigate the bending deformations of the planar thermo-metamaterials, as shown in Figure 2C. The same boundary and loading conditions were used to obtain the bending stiffness and out-of-plane deformations (Figure 2C,i). To simplify the building steps of the numerical model and save the time cost of data set building, we proposed another cantilever bending method, as shown in Figure 2C,ii. More details are provided in Materials and Methods.

Figure 2D,E show the comparisons of the experimental and numerical results for the planar thermo-metamaterials with the square and hexagonal corrugations, respectively. The observed acceptable agreement indicates the accuracy of the numerical models. A comparison of the FE and experimental results for the square thermo-metamaterials is shown in Movie S1, Supporting Information. The overall size of the device, generally determined for a specific implanting area, is influenced by *T*, *H* and *S*. Holding other parameters constant, these parameters particularly affect BS, as shown in Figure 2F–H. Figure 2F indicates that *BS* significantly increases with the increasing *T*. Figure 2G shows that BS initially increases and then decreases as *T* increases within the studied parameter range. Figure 2H demonstrates that BS gradually increases with increasing *S* at larger thicknesses, while at smaller thicknesses, BS first increases and then decreases as *S* increases. Consequently, numerous simulations were conducted regarding these three parameters, enriching the data sets to 720 groups of correspondences between structures and BS.

### AI-Guided Inverse Design for Thermo-Metamaterials Tailored Performance

**Figure 3:**
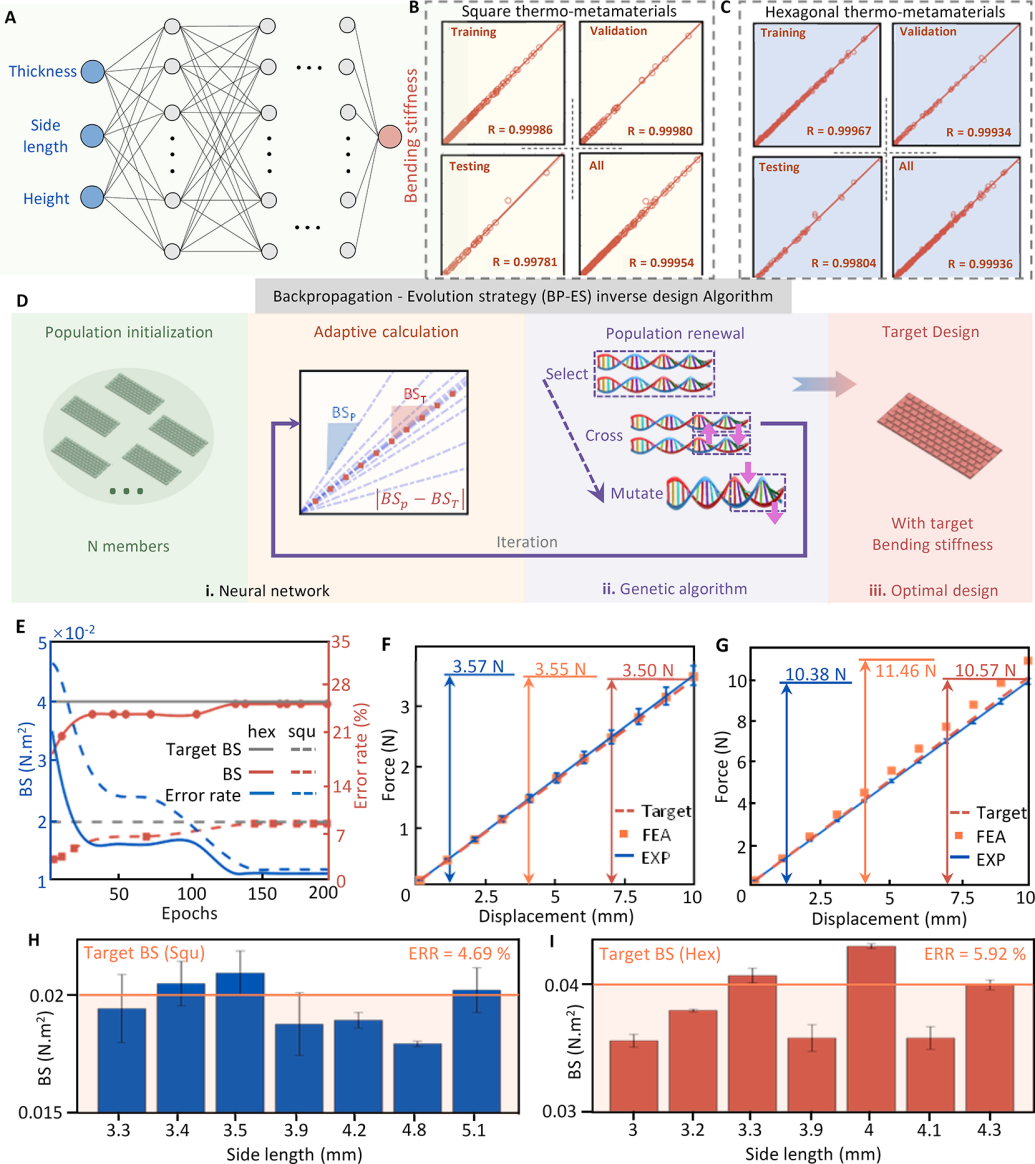
AI inverse design for tailoring the performance of thermo-metamaterials. (A) The architecture of the BP neural networks for predicting the BS of the planar thermo-metamaterials with corrugations. (B) Normalized training, validation and testing results by the AI model for the square thermo-metamaterials. (C) Normalized training, validation and testing results by the AI model for the hexagonal thermo-metamaterials. (D) The proposed AI inverse design process to explore the optimal microstructures for the desired performance (i.e., target BS). (E) Variations of BS and the error rate during the entire evolutionary AI inverse design process. A comparison of the experimental BS values and numerical values for the thermo-metamaterials designs generated by the AI inverse design model: (F) square and (G) hexagonal corrugations. Comparisons and average errors of the *BS* results between the multiple inverse design and experiments for the (H) square and (I) hexagonal thermo-metamaterials.

We first develop a standard AI prediction model using the backpropagation (BP) neural networks. Three variables in the microstructural corrugations, i.e., *T*, *H* and *S*, were selected as the three input neurons in the BP model (Figure 3A). Input neurons start converging to the only output neuron (i.e., BS). The database created in the previous step was used for developing the AI model. A comparison of the normalized predicted and measured BS values for the square and hexagonal plates is shown in Figure 3B,C, respectively. The mean squared errors of the network are 1.86 × 10^–7^ N m^2^ and 5.26 × 10^–7^ for the square and hexagonal thermo-metamaterials, respectively. The well-trained BP model can efficiently predict the BS of the planar thermo-metamaterials with arbitrary corrugations. However, the main challenge for inverse design is achieving desirable bending performance due to the lack of a quantitative relationship between the microstructural parameters and performance. To address this gap, an AI inverse design approach for thermo-metamaterials is developed by combining BP neural networks with the evolution strategy (ES) algorithm,^30^ (Figure 3D). Initially, a total of 300 primordial planar metamaterials were randomly generated by the ES algorithm with varying microstructural parameters. Next, the generated plate structures were fed into the BP neural network to predict their BS. The maladjustment index was calculated by |BS_prediction_ – BS_target_|, representing the absolute value of the difference between the predicted BS and the target BS. This value characterizes how well adapted the individual is to the population in terms of the desired performance; the smaller the value, the more likely the individual’s genes will be passed on to the next generation. Finally, the offspring population was obtained by applying the genetic operations commonly used in the ES process, namely selection, crossover, and mutation within the population. A total of 600 individuals from the offspring population and the parent population of this generation were considered for maladjustment sequencing, and the 300 individuals with the least maladjustment were used as the parent population of the next generation. The termination condition for the inverse design iterative process was achieving an error less than 0.1%, while ensuring that the number of generations was not less than 100.

The variations of BS and error rate during the entire iteration process are shown in Figure 3E. The genetic changes have a higher probability of optimizing the performance of the population in the early stage of the evolutionary process, which leads to the changes of the curves from rapid variation to saturate in the later stages of evolution. Seven square and hexagonal thermo-metamaterial designs generated by the BP-ES inverse design model were randomly selected and printed to compare their BS with the numerical results. In the experimental and numerical model, we guide the output displacement curve by designing a three-point bending condition. In this case, the slope of the force displacement curve is proportional to BS, so the coincidence of the force displacement curve indicates the coincidence of BS. We transform the inverse design of target BS into the inverse design of target force–displacement curve to compare the target force value for a given displacement with that in the experiment and numerical model to display the effect of inverse design more directly. Figure 3F,G display the satisfactory agreement obtained on the target force between the experimental and numerical results. The average error rates are 4.69% and 5.92% for the square and hexagonal thermo-metamaterials, respectively (Figure 3H,I). The experimental results for the square thermo-metamaterials are shown in Movie S2, Supporting Information. Due to the stochastic nature of the initial population generation, the optimal designs obtained by the BP-ES model are not unique (see Table S2 in the Supporting Information).

### AI-Guided Inverse Design of Patient-Specific Deployable Implants

**Figure 4:**
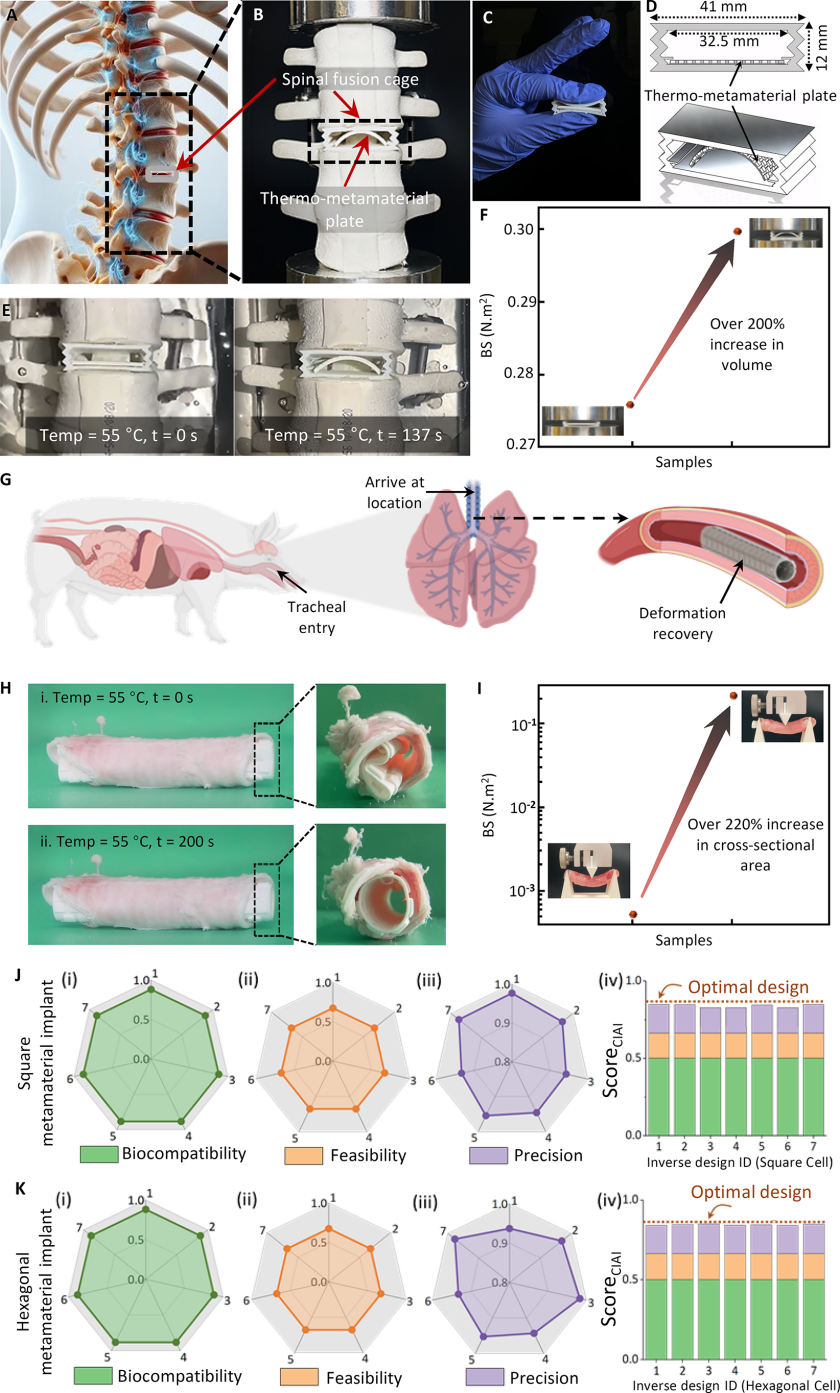
AI inverse design of patient-specific deployable thermo-metamaterial implants. (A) Spinal fusion surgery is performed to permanently connect two or more vertebrae to eliminate motion and treat various conditions. Spinal fusion cages provide critical structural support, withstand significant mechanical loads, and promote bone growth during the fusion process. (B) Implanting a deployable spinal fusion cage in a synthetic biomimetic lumbar spine model at the L3–L4 vertebrae level. (C) Spinal fusion cage in a fully deformed state. (D) Dimensions of the deployable spinal fusion cage. (E) Deployment of the spinal fusion cage. (F) Changes of BS and volume of the fusion cage before and after implant deployment. (G) The procedure for implanting the tracheal stent in porcine models. (H) Deployment of the tracheal stent in a porcine trachea. (I) Changes of BS of tracheal tubes before and after implant deployment. (J) Clinically informed AI inverse design results for the thermo-metamaterials with square corrugations (i) biocompatibility score of seven designs, (ii) feasibility score of seven designs, (iii) precision score of the seven designs, and (iv) practicality score of seven designs to determine the optimal design. (K) Clinically informed AI inverse design results for the thermo-metamaterials with hexagonal corrugations (i) biocompatibility score of seven designs, (ii) feasibility score of seven designs, (iii) precision score of the seven designs, and (iv) practicality score of seven designs to determine the optimal design.

In order to assess the viability of the proposed AI inverse design approach, we devise it to create deployable implants in the form of spinal fusion cages and tracheal stents. We chose these two types of implants because they represent the application of thermo-metamaterials in both planar and tubular forms. Thus, they can provide an acceptable evaluation of the design method across different geometrical and functional requirements. Spinal fusion surgery is a procedure aimed at permanently connecting two or more vertebrae in the spine to eliminate motion between them.^1^ This procedure is often performed to treat conditions such as degenerative disc disease, scoliosis, and spinal instability.^10^ It is crucial for relieving pain, restoring spinal stability, and improving overall function and quality of life in patients suffering from severe spinal disorders.^10^ Spinal fusion cages are critical in providing structural support and stability in the spine after surgery (Figure 4A). These cages must withstand specific mechanical loads and promote bone growth.^31^ Here, we present a deployable spinal fusion cage that is flat during implantation and expands once placed with thermal triggering (Figure 4B–D). Such a deployable spinal implant offers advantages in minimally invasive surgery. The flat form allows for easier and less invasive insertion, reducing the size of the incision needed and minimizing tissue damage (Figure 4C). Once in position, the cage can be recovered to its programmed full size, providing the necessary structural support and stability to the spine.

A synthetic biomimetic lumbar spine model (Sawbones, WA, USA) was used to test the developed deployable cage (Figure 4B). The cage is composed of two parts: a frame made of thermoplastic polyurethane (TPU) and a planar thermo-metamaterial plate made of PLA. The dimensions of the cage implanted at the L3–L4 vertebrae level are shown in Figure 4D. Both TPU and PLA are well-known for their biocompatibility.^32,33^ The *E* of the human disc does not exceed 100 MPa.^34^ Thus, we target this value as the effective *E* of the fusion cage. Using the dimensions of the cage, the moment of inertia will be approximately 2866 mm^4^, resulting in a target BS for the entire deployable cage system, including the TPU frame and buckled PLA plate system, of approximately 0.287 N m^2^. To determine the BS of the plate, we conducted a series of cyclic tests on the cage frame only. With the cage frame providing a BS of 0.275 N m^2^, the thermo-metamaterial plate should provide a BS close to 0.02 N m^2^. Knowing the required BS for the thermo-metamaterial plate, AI inverse design was implemented to find a plate configuration with that *BS*, the procedures of which same as before. The explored design is composed of 98 square corrugated unit cells, with *T*, *H*, and *S* equal to 0.75, 0.6, and 2 mm, respectively. Figure 4E shows how the flat thermo-metamaterial plate restores its programmed shape, achieving a height equivalent to the height of the cage frame after being placed in a bath at 55 °C for 137 s. The cage recovery process is shown in Movie S3, Supporting Information. The flattened cage has a 53% smaller volume compared to its fully deployed state. The cage was then tested under cyclic loading to determine its *BS* when the plate is fully deployed (Movie S4, Supporting Information). The results shown in Figure 4F indicate that the entire cage with its buckled thermo-metamaterial plate offers a BS value of 0.298 N m^2^, which is merely 4% higher than the target BS value. The *E* of the cage and its corresponding *BS* can be tuned to any desired range based on clinician recommendations using the proposed approach.

For the second clinical demonstration, we consider a tracheal stent. These classes of stents are used to keep airways open in patients with conditions such as tracheal stenosis.^35^ Tracheal implants require a balance of mechanical properties. They need to be flexible enough to accommodate natural movement during breathing but also possess sufficient strength to resist collapse and maintain an open airway.^36^ Designing tracheal stents with thermo-metamaterials enables investigating the thermal and mechanical performance in a dynamic environment, where the implant must adapt to physiological movements and temperature variations. This makes tracheal stents a reasonable case study for evaluating the adaptability and functional integration of the designed materials in living tissues. The procedure for implanting the tracheal stent in porcine models is shown in Figure 4G. In this work, we considered designing a thermo-metamaterial tracheal stent for a porcine model (Figure 4H). The corrugated structure of the thermo-metamaterial tracheal stent was designed to achieve a similar diameter to the porcine trachea diameter when fully deployed (diameter = 1.75 mm), as shown in Figure 4H. The designed stent was first flattened at 55 °C, entered through the trachea, then transported to the designated location and secured by the appropriate medical instruments, and finally restored to its original shape by stimulation at 55 °C. The stent was restored to its original shape within 200 s in a water bath at 55 °C. The incision length reduction test, material recovery test and deformation recovery of the tracheal stent in porcine lung in Figures S3–S5 and Movies S5, S6 in Supporting Information. The porcine tracheal stent gained more than 421-fold stiffness enhancement after incorporation (Figure 4I). The cross-sectional area of the stent increased by 223%. The explored design is composed of 98 hexagonal corrugated unit cells, with *T*, *H*, and *S* equal to 0.5, 1, and 3.7 mm, respectively.

### Clinically Informed AI Inverse Design

While focusing solely on the precision of AI-powered inverse design for implants in achieving targeted mechanical properties can be advantageous, it might overlook other crucial aspects. The AI inverse design process can generate multiple parameter sets that deliver the same target performance, such as similar BS values. However, the challenge lies in identifying which designs are most suitable for clinical use in terms of biocompatibility and feasibility. A promising solution to address this issue is developing a clinically informed AI (CIAI) design approach. This approach should integrate biocompatibility, feasibility, and precision simultaneously. By incorporating these factors, the design strategy ensures implants are safe and effective for long-term use, reducing the risks of rejection and complications. Additionally, it improves the practicality of manufacturing and implantation, making advanced treatments more accessible and reliable. Here, we propose a broad vision for the CIAI design approach that prioritizes biocompatibility, feasibility, and precision for successful clinical translation. We demonstrate this vision using corrugated plates as an example. The aim is to pave the way for future development of implants that are both high-performing and clinically viable. The BP neural network-based AI inverse design model can be expressed as
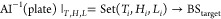
1where AI^–1^ refers to the BP-based AI inverse design model for the planar thermo-metamaterials with respect to the microstructural thickness *T*, height *H*, and side length *L*, and Set(*T*_*i*_,*H*_*i*_,*L*_*i*_) is the parameter sets consisted of series of *T*_*i*_, *H*_*i*_ and *L*_*i*_ that lead to the same target bending stiffness, BS_target_. Taking into account the biocompatibility and feasibility aspects, the CIAI model can be expressed as
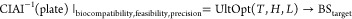
2where, CIAI^–1^ represents the CIAI inverse design model defined in terms of the criteria of biocompatibility, feasibility and precision, and UltOpt(*T*,*H*,*L*) is the optimal parameters for BS_target_. The biocompatibility of an implant is influenced by several factors, such as cytotoxicity (the implant’s potential to harm or kill living cells), stability (chemical stability against corrosion and leaching of harmful chemicals, and long-term mechanical stability), and inflammation (the implant’s potential to trigger an inflammatory response).^37^ Feasibility is also affected by multiple factors, such as manufacturability, implantation difficulty, and cost (including material cost, labor, etc.). Precision is influenced by the configuration of the implant and the mechanical properties of the material used in its design. These parameters can be formulated as follows:
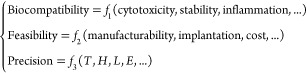
3

The parameters shown in eq 3 represent only a subset of the potential biocompatibility and feasibility-related parameters that are used to simply evaluate the viability in the proposed vision. However, it is possible to assign a weight score to each of these parameters to show how clinically relevant the created designs are. Accordingly, a CIAI practicality score can be defined as
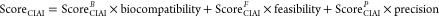
4where Score_CIAI_^*B*^, Score_CIAI_^*F*^ and Score_CIAI_^*P*^ refer to the practicality score of biocompatibility, feasibility, and precision of the implants, respectively. Score_CIAI_^*B*^ and Score_CIAI_^*F*^ are influenced by many “qualitative” factors, as shown in eq 3, subjective to user opinion. This issue can be addressed using a fuzzy evaluation method like the fuzzy analytic hierarchy process (AHP),^38^ which enables converting qualitative measures to quantitative scoring of biocompatibility and feasibility within the CIAI inverse design model. AHP is a structured decision-making approach grounded in mathematics and psychology. Fuzzy AHP extends the classical AHP method by incorporating the decision-maker’s uncertainty.^38,39^ To demonstrate the feasibility of fuzzy AHP for determining Score_CIAI_^*B*^, Score_CIAI_^*F*^ and Score_CIAI_^*P*^, a simple experiment was conducted. Ten clinical surgeons were asked to grade the importance of biocompatibility, feasibility, and precision of the implants on a scale of 0–10, resulting in a 3 × 3 matrix (see Materials and Methods). Subsequently, the fuzzy AHP method was employed to determine practicality scores. Based on this analysis, Score_CIAI_^*B*^, Score_CIAI_^*F*^ and Score_CIAI_^*P*^ were calculated to be 0.57, 0.24, and 0.19, respectively.

Thus, eq 4 can be expressed as
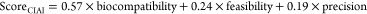
5

The same fuzzy AHP method can be used to score the chosen factors impacting biocompatibility and feasibility (see eq 3). The analysis for this case study is presented in Note S1, Supporting Information. Accordingly, we have
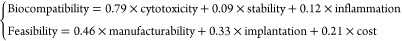
6

The weights for each factor shown in eq 6 are normalized to range from 0.1 to 0.9, with 0.1 representing the lowest value and 0.9 indicating the highest. For example, a weight of 0.1 for cytotoxicity signifies that the material used has the potential to harm cells, while a weight of 0.9 implies no cytotoxicity. The precision score can be calculated by comparing the predicted *BS* with given *T*, *H*, *S*, E and the target BS as
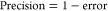
7where error is . Substituting eqs 6 and 7 into eq 5, the practicality score Score_Pract_ is obtained as
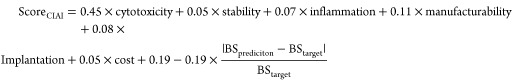
8

The AI inverse design plays a crucial role in the CIAI approach by enabling the customization of precision based on other clinically relevant factors. For example, the precision for the sixth square thermo-metamaterial implant (Table S3 in the Supporting Information) is 0.93. Clinical surgeons consulted in this work assigned values of 0.9, 0.7, 0.9, 0.5, 0.9, and 0.7 to cytotoxicity, stability, inflammation, manufacturability, implantation difficulty, and cost, respectively. Using eq 8, the Score_CIAI_ for this design is calculated to be 0.84. If clinicians prioritize maintaining the same biocompatibility and feasibility levels but desire a higher Score_CIAI_, AI inverse design can be employed to explore the design space for increased precision. In this scenario, the seventh square thermo-metamaterial implant (Table S3 in the Supporting Information) with *T*, *H*, *S*, and *E* values of 1.15, 1.49, 5.12 mm, and 2400 MPa emerges as a potential optimal design, offering a precision score of 0.97 and a Score_CIAI_ of 0.85. Assuming the same set of biocompatibility and feasibility factors for all square and hexagonal plates in this study, their calculated Score_CIAI_ are shown in Figure 4J,K respectively. More analysis details are presented in Tables S3 and S4 in the Supporting Information.

A limitation of this approach is that the assigned weights are still subjective to experts’ opinion. However, such an approach is a common practice in many clinical fields that rely on clinician judgment. For instance, the widely used Mirels’ score incorporates multiple qualitative factors weighted by clinicians to determine the degree of cortical destruction solely based on radiographic images.^40^ In practice, it is possible to determine the weights for parameters such as cytotoxicity, stability and inflammation from laboratory testing of the materials used in the implants compared to a controlled material widely used in implant design, such as titanium. Given the novelty of this approach, it is crucial to establish a standardized protocol. This can be achieved by considering a substantially larger group of clinicians and developing a range of acceptable Score_CIAI_ values. These values should encompass not only the limited parameters included in eq 3 but also account for various implant types like those used in cardiac stents, orthopedic implants, and so on.

## Conclusion

In this paper, we introduced a novel approach for designing deployable implants using an AI-powered inverse design paradigm. The created implants leverage thermo-metamaterials that exhibit tunable mechanical properties in response to temperature changes. This enables minimally invasive surgery through a small incision and subsequent deployment to the desired functional shape within the body. The core of our approach is an AI inverse design model that integrates an evolutionary algorithm with a neural network. This model automatically determines the optimal microstructural parameters for thermo-metamaterials to achieve desired mechanical properties, such as BS. The effectiveness of this approach is validated through the design of patient-specific spinal fusion cages and tracheal stents. Our results demonstrate that deployable thermo-metamaterial implants can achieve significant increases in volume or cross-sectional area upon deployment. Beyond achieving the desired mechanical properties, we present a broader vision for a CIAI design process. This process prioritizes biocompatibility, feasibility, and precision for successful clinical translation. The proposed fuzzy AHP method is a potential tool for incorporating these crucial factors into the design process, generating practicality scores for various designs. This CIAI design process can potentially pave the way for developing high-performing and clinically viable implants tailored to individual patient needs. Future work should focus on establishing standardized protocols for the CIAI design process and conducting in vivo studies to evaluate the long-term performance of these innovative thermo-metamaterial implants. Additionally, optimizing deployment mechanisms can improve the predictability and control of the shape memory behavior of the implants. Investigating new biocompatible materials with enhanced properties, such as biodegradability, higher mechanical strength, and improved thermal responsiveness, is also crucial.

## Materials and Methods

### 3D Modeling

The corrugated metasurfaces were first parametrically modeled using Rhino 7 software by entering two parameters, side length and height, to obtain a surface. The model is then stored as a 3dm file using the Grasshopper plug-in, and imported into Solidworks 2020. A downward surface thickening operation was considered to create 3D printable plate models. For the stent, the bending function was introduced on top of the plate, and the stent model was obtained by setting the bending angle to 360 deg.

### Numerical Model

The commercial software ABAQUS was used to build numerical models. For the model in Figure 2C,i three rigid shell models were established through extrusion, and then planar metamaterial model was imported from Solidworks and assembled. The contact between the individual components was the universal contact from surface to surface. The tangential behavior penalty was selected. The friction coefficient was set to 0.3, with hard contact selected for normal behavior. A static general approach was used for the analysis step, and C3D10 was chosen for the grid type. The setup in Figure 2C,ii remains consistent with the above parameters, except for a modification in the loading method. Despite this change, the results obtained were nearly identical.

### 4D Printing

The printing consumables used in this paper are from Anycubic Company. The stl model file of the corrugated metasurfaces was imported into ideaMaker 4.3.3 software for slicing. The automatic repair was performed first, the fill rate was selected 100%. No support structure was used and only the Brim base plate was attached. The sliced file was imported into the printer RAISE3D Pro2, using only a single side nozzle, a print speed of 35.0 mm/s, a wire diameter of 1.75 mm, a print temperature of 230 °C, a hot bed temperature of 60 °C, and an experimentally measured *E* of 2400 MPa.

### Training of the Neural Network

In order to achieve an effective accuracy for the neural network, for both square and hexagon cells, we performed 480 sets of structural design and FEA calculations, respectively. We divided the data set into training, validation and test sets in the ratio of 8:1:1 and used MATLAB software and Intel Core i7-9700 CPU @ 3.00 GHz for computation. The optimal BP models were built with 5 hidden layers, every hidden layer with 15 neurons.

### Evolutionary Strategy Details

The number of initial population was set to 300. We used three genetic operations for the evolutionary method: selection, crossover and mutation. For selection, we used the tournament selection method to randomly select two individuals from the parent population. For crossover, we used the single-point crossover method, where two individuals are first selected randomly from the parent population, then the location of the crossover point is randomly selected, and finally the genes are exchange at the crossover point. The crossover probability was set at 0.8 to speed up population evolution. For mutation, we used a single point mutation method. The mutation probability was set at 0.2 to prevent a locally optimal outcome. The ES algorithm was combined with neural network to form inverse design algorithm. The codes in this paper were implemented in MATLAB.

## Data Availability

The data that support the findings of this study are available from the corresponding author upon reasonable request.

## Supporting Information Available

Determination of the CIAI practicality scores; comparison of the bending response of corrugated plates and plain plates; 3D printing process of corrugated plates and implants; incision length reduction test; material change test before and after shape memory cycling; deployable tracheal stent in porcine lung; structural parameters of the thermo-metamaterial implants explored during the AI inverse design process; clinically informed AI inverse design analysis for square and hexagonal thermo-metamaterial implants; the error limits (the maximum error) of certain experimental data employed in this work (PDF)
Verification of the finite element analysis results (MP4)
Experimental testing of bending stiffness for the AI-designed thermo-metamaterial plates (MP4)
The AI-designed thermo-metamaterial spinal fusion cage under uniaxial loading (MP4)
Deformation recovery of the thermo-metamaterial spinal fusion cage (MP4)
Deformation recovery of the thermo-metamaterial tracheal stent in porcine trachea (MP4)
Deformation recovery of the thermo-metamaterial tracheal stent in porcine lung (MP4)
