# Supplementary material for: Artificial Intelligence-Guided Inverse Design of Deployable Thermo-Metamaterial Implants
Source: ACS Appl Mater Interfaces. 2025 Jan 2;17(2):2991–3001. doi: 10.1021/acsami.4c17625 (PMC11744508; doi:10.1021/acsami.4c17625)
Supplement: Supplementary file 1 — am4c17625_si_001.pdf [file am4c17625_si_001.pdf]

Supporting Information

Artificial Intelligence-Guided Inverse Design of Deployable Thermo-Metamaterial Implants

Pengcheng Jiao[1,*], Chenjie Zhang[1], Wenxuan Meng[2,3], Jiajun Wang[1], Daeik Jang[4], Zhangming Wu[5], Nitin Agarwal[6,7,8], Amir H. Alavi[2,3,4,*]

[1]Ocean College, Zhejiang University, Zhoushan 316021, China

[2]Department of Bioengineering, University of Pittsburgh, Pittsburgh, PA 15261, USA

[3]Department of Mechanical Engineering and Materials Science, University of Pittsburgh, Pittsburgh, PA 15261, USA

[4]Department of Civil and Environmental Engineering, University of Pittsburgh, Pittsburgh, PA 15261, USA

[5]College of Engineering, Cardiff University, Cardiff, CF10 3AT, UK

[6]Department of Neurological Surgery, University of Pittsburgh School of Medicine, Pittsburgh, PA 15213, USA

[7]Department of Neurological Surgery, University of Pittsburgh Medical Center, Pittsburgh, PA 15213, USA

[8]Neurological Surgery, Veterans Affairs Pittsburgh Healthcare System, Pittsburgh, PA 15240, USA

[*]Corresponding authors: pjiao@zju.edu.cn (P.J) and alavi@pitt.edu (A.H.A).

Note S1: Determination of the CIAI practicality scores

Fuzzy mathematics is used to handle situations where data has unclear boundaries or is difficult to quantify precisely. This method allows the model to incorporate physical factors from the real world into the AI inverse design process. The resulting model, called CIAI, presumably combines these elements (Clear, Inverse, and Analogical) for design. The specific steps are as follows:

Step 1: Determine the set of factors that influence the evaluation results

The $\mathrm{Score_{CIAI}}$ practicality score is used to define a standard protocol for the applicability of implantable corrugated plates. This score can be used to obtain the final optimal designs. The score is affected by many factors. We chose only three main factors for this demonstration: biocompatibility, feasibility and precision. Biocompatibility is affected by cytotoxicity (the implant's potential to harm or kill living cells), stability (chemical stability against corrosion and leaching of harmful chemicals, and long-term mechanical stability), and inflammation (the implant's potential to trigger an inflammatory response). Feasibility is affected by multiple factors, such as manufacturability, implantation difficulty, and cost (including material cost, labor, etc.). Precision is influenced by the configuration of the implant and the mechanical properties of the material used in its design.

Step 2: Calculate the weight of scores at each level

We introduced analytic hierarchy process (AHP),[1,2] to determine the weights. To assess surgeon priorities, ten surgeons were asked to score biocompatibility, feasibility, and precision on a 0-10 scale. The lower scores signified higher importance. The scoring system results in a 3 by 3 matrix shown in Table S1. The values in row *i* and column *j* represented the importance of the *i* element compared with the *j* element. $F_{i,j}$ in Eq. (1) represents the set of factors considered by the jth weight matrix at level *i*, where the ordering of elements is consistent with the rank and column ordering of the weight matrix.

Table S1. The scores assigned to biocompatibility, feasibility, and precision by surgeons.

**Factor**	Biocompatibility	Feasibility	Precision
Biocompatibility	1	3	4
Feasibility	1/3	1	2
Precision	1/4	1/2	1

$$F_{1,1} = (Biocompatibility, Feasibility, Precision) \quad (1)$$

Due to the symmetry of the matrix, as shown in Table S1, experts only need to provide the three scores highlighted in red. The remaining elements can be calculated using their reciprocals. The example shown in Table S1 suggests that biocompatibility is the most important parameter to the surgeons, followed by feasibility and then precision. We averaged the scores of ten experts and formed the weight matrix A.

$$A = \begin{pmatrix} 1 & 2.376 & 3.024 \\ 0.421 & 1 & 1.212 \\ 0.331 & 0.825 & 1 \end{pmatrix} \quad (2)$$

By normalizing the scores, we have:

$$A_{norm} = \begin{pmatrix} 0.571 & 0.566 & 0.578 \\ 0.240 & 0.238 & 0.231 \\ 0.189 & 0.196 & 0.191 \end{pmatrix} \quad (3)$$

We use $\omega_{i,j}$ to represent the jth weight vector of level *i*. After averaging each row of A_{norm}, we obtain:

$$\omega_{1,1} = (0.57 \quad 0.24 \quad 0.19) \quad (4)$$

Accordingly, $\mathrm{Score_{CIAI}}$ can be calculated as:

$$\mathrm{Score_{pract}} = 0.57\times\mathrm{Biocompat} + 0.24\times\mathrm{Feasibility} + 0.19\times\mathrm{Precision} \tag{5}$$

The next step is to determine the first weight vector of the second level, i.e. biocompatibility factors:

$$F_{2,1} = (\mathrm{Cytotoxicity,\ Stability,\ Inflammation}) \tag{6}$$

Averaging the scores of ten experts, we have:

$$B = \begin{pmatrix} 1 & 8.251 & 6.729 \\ 0.121 & 1 & 0.778 \\ 0.149 & 1.286 & 1 \end{pmatrix} \tag{7}$$

By normalizing the scores, we have:

$$B_{norm} = \begin{pmatrix} 0.788 & 0.783 & 0.791 \\ 0.095 & 0.095 & 0.091 \\ 0.117 & 0.122 & 0.118 \end{pmatrix} \tag{8}$$

After averaging each row of B_{norm}, we obtain:

$$\omega_{2,1} = (0.79 \quad 0.09 \quad 0.12) \tag{9}$$

Thus, biocompatibility can be expressed as:

$$\mathrm{Biocompatibility} = 0.79\times\mathrm{Cytotoxicity}\ +0.09\times\mathrm{Stability} + 0.12\times\mathrm{Inflammation} \tag{10}$$

The next step is to determine the second weight vector of the second level, i.e. feasibility:

$$F_{2,2}=(\mathrm{Manufacturability,Implantation\ ,Cost}) \tag{11}$$

Averaging the scores of ten experts, we have:

$$C = \begin{pmatrix} 1 & 1.348 & 2.197 \\ 0.742 & 1 & 1.523 \\ 0.455 & 0.657 & 1 \end{pmatrix} \tag{12}$$

By normalizing the scores, we obtain:

$$C_{norm} = \begin{pmatrix} 0.455 & 0.449 & 0.465 \\ 0.338 & 0.333 & 0.323 \\ 0.207 & 0.219 & 0.212 \end{pmatrix} \tag{13}$$

After averaging each row of C_{norm}, we obtain:

$$\omega_{2,2} = (0.46 \quad 0.33 \quad 0.21) \tag{14}$$

Thus, feasibility can be expressed as:

$$\mathrm{Feasibility} = 0.46\times\mathrm{Manufacturability}\ +0.33\times\mathrm{Implantation}\ + 0.21\times\mathrm{Cost} \tag{15}$$

Finally, the third weight vector of the second level, i.e. precision, should be determined. For this indicator, we use a more objective algorithm to obtain its value. The precision score can be calculated by

$$Precision = 1 - error \tag{16}$$

where, *error* represents $\frac{|BS_{prediciton} - BS_{target}|}{BS_{target}}$. Thus, $\mathrm{Score_{CIAI}}$ can be expressed as:

$$\mathrm{Score_{CIAI}} = 0.45\times\mathrm{Cytotoxicity}\ + 0.05\times\mathrm{Stability} + 0.07\times\mathrm{Inflammation}\ + 0.11\times\mathrm{Manufacturability} + 0.08\times \mathrm{Implantation}\ +0.05\times\mathrm{Cost} + 0.19 - 0.19\times\frac{|\mathrm{BS_{prediciton}} - \mathrm{BS_{target}}|}{\mathrm{BS_{target}}} \tag{17}$$

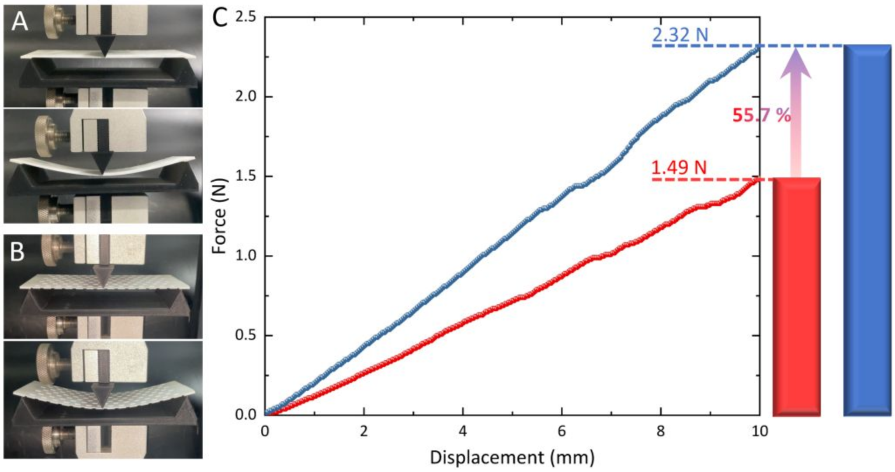

Figure S1. Comparison of the bending response of corrugated plates and plain plates (both 0.9 mm thick). (**A**) Three-point bending test of the plain plate. (**B**) Three-point bending test of the corrugated plate. (**C**) Comparison of force displacement curves.

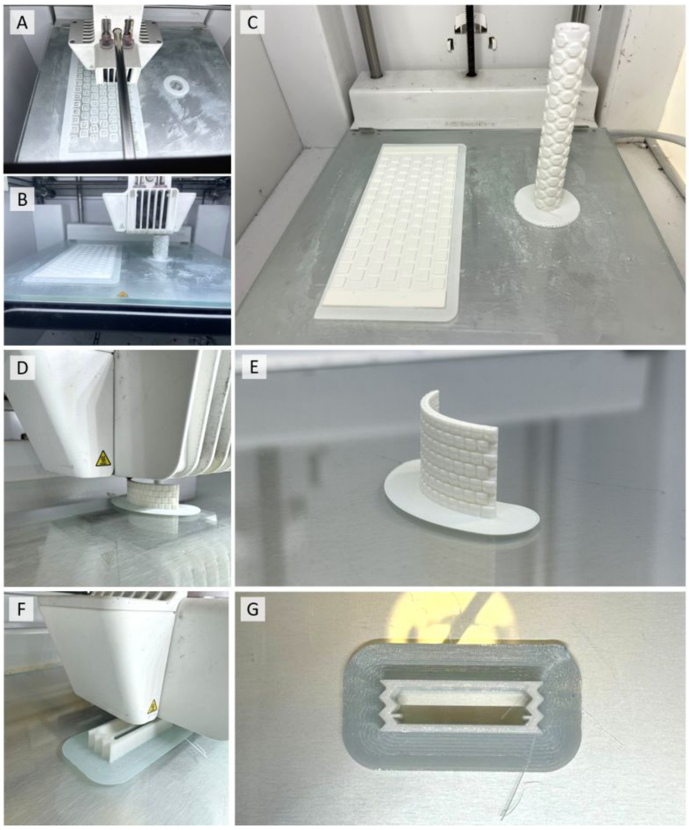

Figure S2. 3D printing process of corrugated plates and implants. (**A**) 3D printing of the bottom support for the corrugated plate. (**B**) 3D printing of the corrugated stent. (**C**) 3D printed stent. (**D**) Printing process of the curved plate for spinal fusion. (**E**) 3D printed curved plate for spinal fusion. (**F**) 3D printing of the cage frame. (**G**) 3D printed spinal fusion cage frame.

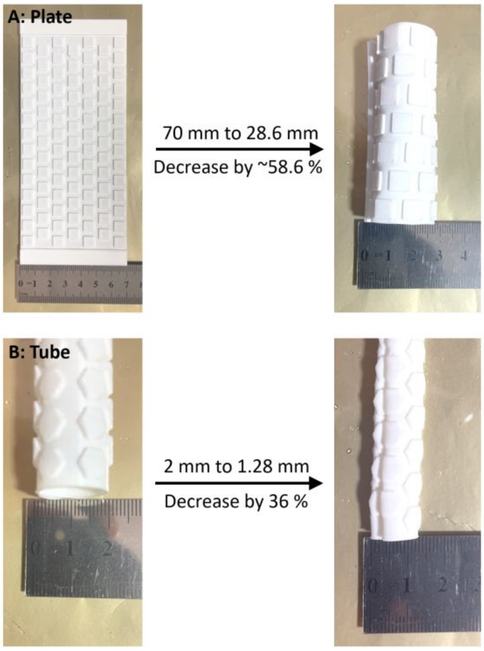

Figure S3. Incision length reduction test. (**A**) For the plate, the incision length decreased from 70 mm to 28.6 mm. (**B**) For the stent, the incision length decreased from 2 mm to 1.28 mm.

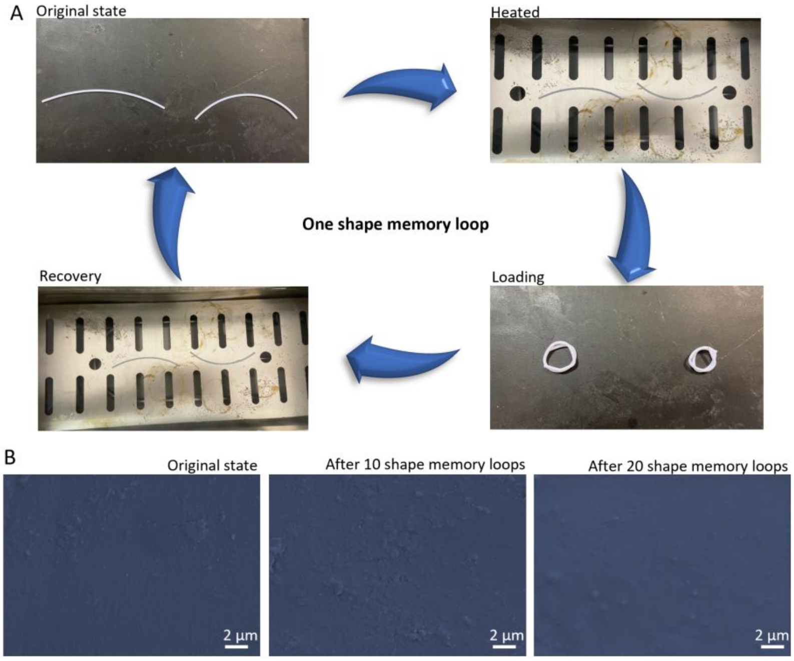

Figure S4. Material change test before and after shape memory cycling. (**A**) The loading process of the shape memory loop is as follows: First, the printing wire is placed in a 55 °C high temperature water bath for 5 minutes. It is then taken out and quickly rolled into a hoop, cooled for 5 minutes, and placed in 55 °C high temperature water bath to restore the shape of the wire. Finally, it it is cooled to room temperature. (**B**) Scanning electron microscopy (SEM) results show that after multiple shape memory cycles, the printed wires indicate no significant cracks.

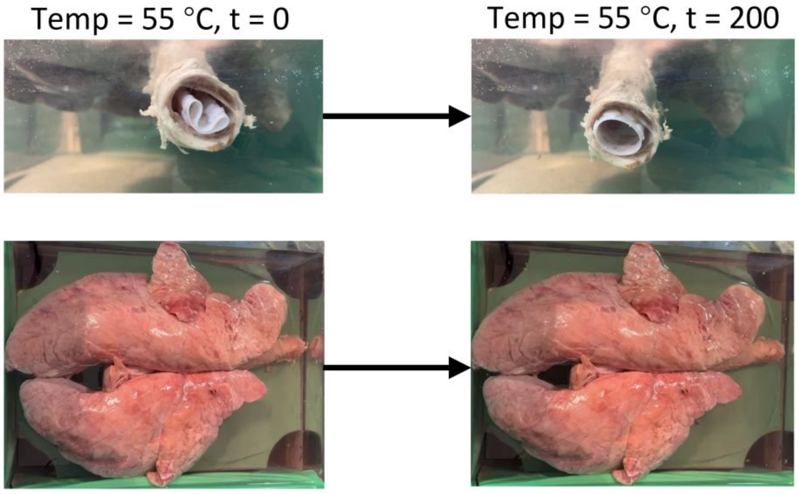

Figure S5. Deployable tracheal stent in porcine lung. The programmed corrugated stent was placed in a 55 °C water bath. After 200 seconds, the stent recovered its original shape.

Table S2. Structural parameters of the thermo-metamaterial implants explored during the AI inverse design process.

ID	Side length (mm)	Thickness (mm)	Height (mm)
	Square		
1	3.30	1.22	2.52
2	3.40	1.20	1.00
3	3.51	1.26	0.96
4	3.94	1.25	0.68
5	4.20	1.20	0.50
6	4.84	1.18	2.89
7	5.12	1.15	1.49
	Hexagonal		
1	3.11	1.33	3.35
2	3.27	1.45	2.95
3	3.30	1.40	2.20
4	3.92	1.28	3.30
5	4.36	1.38	1.51
6	4.10	1.50	0.60
7	4.31	1.35	1.29

Table S3. Clinically-informed AI inverse design analysis for square thermo-metamaterial implants.

Designs	1	2	3	4	5	6	7
Cytotoxicity	0.9	0.9	0.9	0.9	0.9	0.9	0.9
Stability	0.7	0.7	0.7	0.7	0.7	0.7	0.7
Inflammation	0.9	0.9	0.9	0.9	0.9	0.9	0.9
Manufacturability	0.5	0.5	0.5	0.5	0.5	0.5	0.5
Implantation difficulty	0.9	0.9	0.9	0.9	0.9	0.9	0.9
Cost	0.7	0.7	0.7	0.7	0.7	0.7	0.7
S (mm)	3.30	3.40	3.51	3.94	4.20	4.84	5.12
T (mm)	1.22	1.20	1.26	1.25	1.20	1.18	1.15
H (mm)	2.52	1.00	0.96	0.68	0.50	2.89	1.49
E (MPa)	2400	2400	2400	2400	2400	2400	2400
Error	0.02	0.03	0.06	0.05	0.05	0.07	0.03
Biocompatibility	0.88	0.88	0.88	0.88	0.88	0.88	0.88
Feasibility	0.68	0.68	0.68	0.68	0.68	0.68	0.68
Precision	0.98	0.97	0.94	0.95	0.95	0.93	0.97
**Score**	**0.85**	**0.85**	**0.84**	**0.84**	**0.85**	**0.84**	**0.85**

Table S4. Clinically-informed AI inverse design analysis for hexagonal thermo-metamaterial implants.

Designs	1	2	3	4	5	6	7
Cytotoxicity	0.9	0.9	0.9	0.9	0.9	0.9	0.9
Stability	0.7	0.7	0.7	0.7	0.7	0.7	0.7
Inflammation	0.9	0.9	0.9	0.9	0.9	0.9	0.9
Manufacturability	0.5	0.5	0.5	0.5	0.5	0.5	0.5
Implantation difficulty	0.9	0.9	0.9	0.9	0.9	0.9	0.9
Cost	0.7	0.7	0.7	0.7	0.7	0.7	0.7
S (mm)	3.11	3.27	3.30	3.92	4.36	4.10	4.31
T (mm)	1.33	1.45	1.40	1.28	1.38	1.50	1.35
H (mm)	3.35	2.95	2.20	3.30	1.51	0.60	1.29
E (MPa)	2400	2400	2400	2400	2400	2400	2400
Error	0.06	0.03	0.02	0.06	0.05	0.07	0.02
Biocompatibility	0.88	0.88	0.88	0.88	0.88	0.88	0.88
Feasibility	0.68	0.68	0.68	0.68	0.68	0.68	0.68
Precision	0.94	0.97	0.98	0.94	0.95	0.93	0.98
**Score**	0.84	0.85	0.85	0.84	0.85	0.84	0.85

Table S5. The error limits (the maximum error) of certain experimental data employed in this work.

Experiment name	Maximum error	Maximum error/experimental value
Accuracy verification experiment of numerical simulation results (square unit cells)	0.15	3.7%
Accuracy verification experiment of numerical simulation results (hexagonal unit cells)	0.27	5.3%
Accuracy verification experiment of inverse design results (square unit cells)	0.001372	7.3%
Accuracy verification experiment of inverse design results (hexagonal unit cells)	0.001083641	3.0%

Movies

Movie S1 (.MP4 Format). Verification of the FE results.

Movie S2 (.MP4 Format). Experimental testing of *BS* for the AI-designed square thermo-metamaterial plates.

Movie S3 (.MP4 Format). The AI-designed thermo-metamaterial spinal fusion cage under uniaxial loading.

Movie S4 (.MP4 Format). Deformation recovery of the thermo-metamaterial spinal fusion cage.

Movie S5 (.MP4 Format). Deformation recovery of thermo-metamaterial tracheal stent in porcine trachea.

Movie S6 (.MP4 Format). Deformation recovery of thermo-metamaterial tracheal stent in porcine lung.

References

1. Leung, L. C., & Cao, D. (2000). On consistency and ranking of alternatives in fuzzy AHP. European journal of operational research, 124(1), 102-113.
2. Emrouznejad, A., & Ho, W. (2017). Fuzzy analytic hierarchy process. CRC Press.
